# A Murine *Myh6^MerCreMer^* Knock-In Allele Specifically Mediates Temporal Genetic Deletion in Cardiomyocytes after Tamoxifen Induction

**DOI:** 10.1371/journal.pone.0133472

**Published:** 2015-07-23

**Authors:** Jianyun Yan, Lu Zhang, Nishat Sultana, David S. Park, Akshay Shekhar, Lei Bu, Jun Hu, Shegufta Razzaque, Chen-Leng Cai

**Affiliations:** 1 Department of Developmental and Regenerative Biology, The Black Family Stem Cell Institute, and The Mindich Child Health and Development Institute, Icahn School of Medicine at Mount Sinai, New York, NY, United States of America; 2 Leon H. Charney Division of Cardiology, New York University School of Medicine, New York, NY, United States of America; University of Cincinnati, College of Medicine, UNITED STATES

## Abstract

A mouse model that mediates temporal, specific, and efficient myocardial deletion with Cre-LoxP technology will be a valuable tool to determine the function of genes during heart formation. *Mhy6* encodes a cardiac muscle specific protein: alpha-myosin heavy chain. Here, we generated a new *Myh6-MerCreMer* (*Myh6^MerCreMer/+^*) inducible Cre knock-in mouse by inserting a *MerCreMer* cassette into the *Myh6* start codon. By crossing knock-in mice with *Rosa26* reporter lines, we found the *Myh6^MerCreMer/+^* mice mediate complete Cre-LoxP recombination in cardiomyocytes after tamoxifen induction. X-gal staining and immunohistochemistry analysis revealed that *Myh6*-driven Cre recombinase was specifically activated in cardiomyocytes at embryonic and adult stages. Furthermore, echocardiography showed that *Myh6^MerCreMer/+^* mice maintained normal cardiac structure and function before and after tamoxifen administration. These results suggest that the new *Myh6^MerCreMer/+^* mouse can serve as a robust tool to dissect the roles of genes in heart development and function. Additionally, myocardial progeny during heart development and after cardiac injury can be traced using this mouse line.

## Introduction

Congenital heart disease (CHD) is the leading cause of birth defects in humans [1–3] with an incidence varying from 19 to 75 per 1,000 of live births [4]. Deletion of genes through Cre-LoxP technology in mice has facilitated the discovery of a number of genes critical for heart development and function (e.g., *Nkx2*.*5*, *Hand2*, *Gata4*, *Mef2c*, *Tbx5* and *Tbx20*) [5–10]. To further understand the mechanisms underlying heart development and disease, several inducible Cre mouse lines with *tTA/rtTA/TetO* and *MerCreMer* (Cre recombinase fused to two mutated estrogen receptor (Mer) ligand binding domains) have been developed and allow myocardial specific deletion of genes of interest in a temporal manner [11–13]. However, the *tTA/rtTA/TetO* animal models do not mediate instant and complete Cre excision [14], and the transgenic *α-MHC-MerCreMer* mice are imperfect deleter since they display cardiac functional defects after tamoxifen treatment [15–21]. These unfavorable features may eventually lead to misinterpretation of data in cardiac studies.

*Myh6* (*α-MHC*, *MYHC* and *MYHCA*) encodes the cardiac muscle specific protein alpha-myosin heavy chain and is critical for heart development [22–24]. *MYH6* mutations in humans cause atrial septal defect as well as dilated and hypertrophic cardiomyopathy [22–24]. The alpha-myosin heavy chain is dynamically expressed in cardiomyocytes during heart formation [25]. In this study, we created *Myh6* ^*MerCreMer/+*^ knock-in mice by inserting the *MerCreMer* cassette into the *Myh6* start codon. *Myh6*-driven Cre recombinase was specifically activated in cardiomyocytes after tamoxifen induction at embryonic and adult stages. Thus, the *Myh6* ^*MerCreMer/+*^ knock-in mouse model may be a useful instrument in the temporal genetic deletion of genes of interest in cardiomyocytes in addition to tracing myocardial lineage during development and after cardiac injury.

## Materials and Methods

### Animals

**Fig 1:**
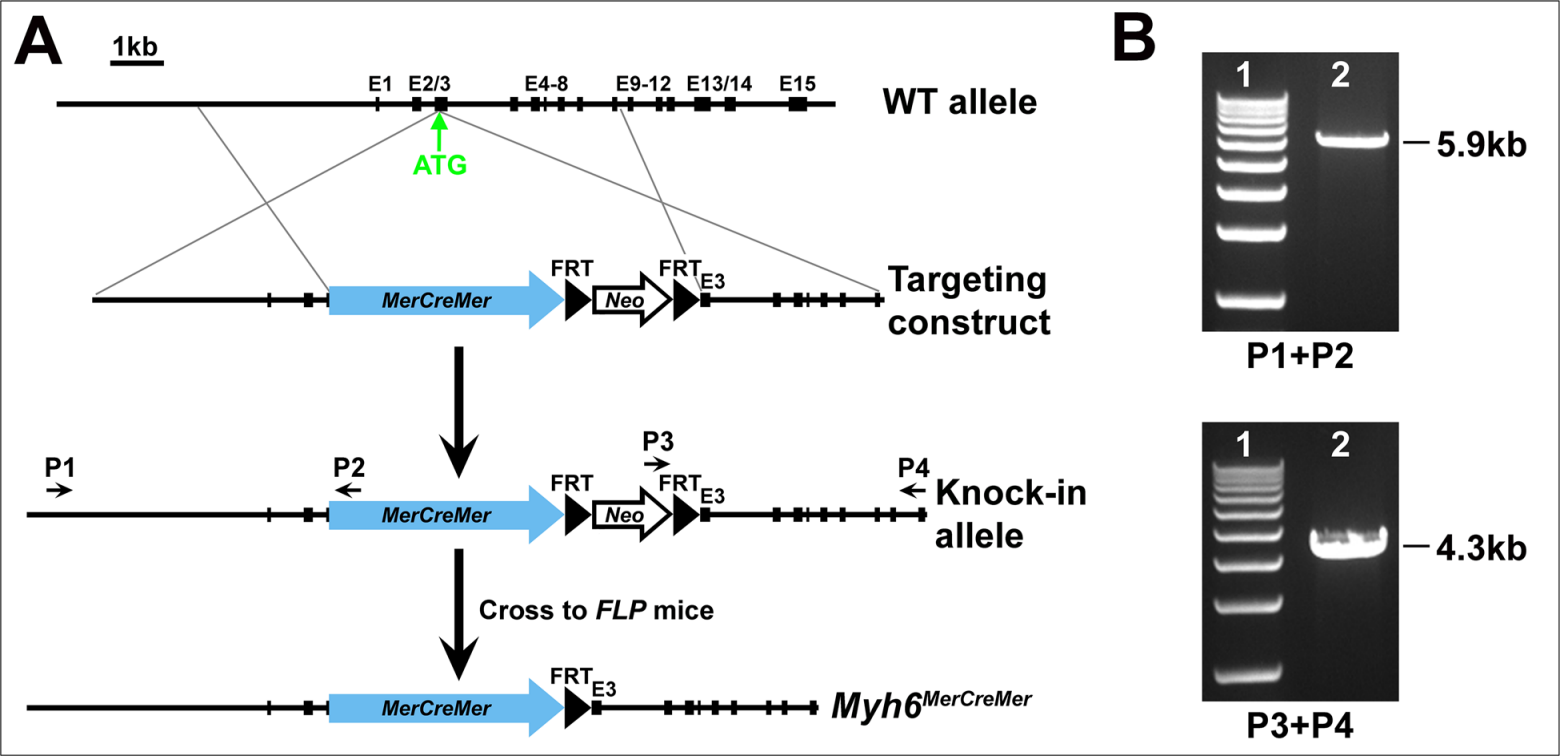
Generation of *Myh6* ^*MerCreMer/+*^ knock-in mouse. **(A)** Schematic diagram of gene targeting. The *MerCreMer-FRT-Neo-FRT* cassette was inserted into the *Myh6* locus (6 bp upstream ATG). The *Neo* cassette was removed by crossing *Myh6* ^*MerCreMer-Neo/+*^ mice with the Flippase deleter (*FLPe*) mice. **(B)** Long-range PCR of genomic DNA from the positive ES cells generated a 5.9-kb recombinant fragment at 5' end and a 4.3-kb recombinant fragment at 3' end with primers P1/P2 and P3/P4, respectively.

*Myh6* ^*MerCreMer/+*^ knock-in mice were generated by gene targeting. A *MerCreMer-FRT-Neo-FRT* cassette was inserted 6 bp upstream of the start codon of *Myh6* (with disruption of endogenous ATG). The targeting construct contains a *MerCreMer-FRT-Neo-FRT* cassette flanked by 5' and 3' homologous arms (Fig 1). A linearized construct was transfected into mouse embryonic stem (ES) cells. Positive ES cells were identified by long-range PCR (Roche) with a primer external to the homologous arms and a primer located in the *MerCreMer-FRT-Neo-FRT* cassette. PCR fragments were verified by DNA sequencing.

Chimeric mice derived from the positive ES cells were crossed with Black Swiss wild type mice to obtain *Myh6* ^*MerCreMer-Neo/+*^ mice. The *Myh6* ^*MerCreMer/+*^ allele was obtained by crossing *Myh6* ^*MerCreMer-Neo/+*^ mice with *FLPe* mice [26]. *R26R* ^*lacZ/+*^ and *R26R* ^*tdTomato/+*^ mice were obtained from the Jackson Laboratory [27, 28]. *Myh6* ^*MerCreMer/+*^ mice were genotyped by PCR of DNA isolated from mouse tails using the following primers: GCAGGCACTTTACATAGAGTCCTG (Forward, 5'→3'); GTTCAGCATCCAACAAGGCACTGA (Reverse, 5'→3'). Mice were euthanized through cervical dislocation for collecting embryonic and postnatal tissues. Animal husbandry procedures were approved by the Institutional Animal Care and Use Committee at Icahn School of Medicine at Mount Sinai (LA09-00494) and are in compliance with NIH guidelines (PHS Animal Welfare Assurance A3111-01).

### Tamoxifen Administration

*Myh6* ^*MerCreMer/+*^ mice were crossed with *R26R* ^*lacZ/+*^ or *R26R* ^*tdTomato/+*^ reporter mice to get *Myh6* ^*MerCreMer/+*^;*R26R* ^*lacZ/+*^ and *Myh6* ^*MerCreMer/+*^;*R26R* ^*tdTomato/+*^ doubly heterozygous animals. Tamoxifen (Sigma-Aldrich) was prepared in sesame oil (Sigma-Aldrich) and was administered to the pregnant (0.05 mg/g body weight/day) and adult (0.1 mg/g body weight/day) mice by intraperitoneal injections for 2 (for embryonic) or 3 (for postnatal) consecutive days [12, 21, 29]. Tissues were harvested 24 hours after the final administration of Tamoxifen for X-gal staining or immunofluorescence.

### X-Gal and Trichrome Staining

Whole mouse embryos and hearts were harvested at the indicated time points. β-galactosidase activity was assessed by X-gal staining as described previously [30]. Mouse embryos or hearts were dissected in PBS and fixed in 4% paraformaldehyde/PBS for 30 min at 4°C. The fixed samples were then washed twice with PBS, followed by staining in X-gal solution overnight at room temperature [31]. For cryosections, cardiac samples were embedded in Optimal Cutting Temperature (OCT) compound (Tissue-Tek) on dry ice and were cut to 10 μm in thickness. Frozen sections were stained in X-gal solution at 37°C overnight. After two washes in PBS, cardiac sections were mounted in permount medium and the images were then captured under a Leica microscope. Cardiac fibrosis was examined by Masson’s trichrome Stain Kit (Sigma-Aldrich) using the manufacturer’s protocol.

### Immunofluorescence

Mouse hearts were fixed in 4% paraformaldehyde/PBS on ice for 30 min. They were then embedded in OCT using standard procedure. Cryosections were cut to 6 μm in thickness for immunofluorescence. Tissue sections were washed with PBS and blocked with 10% goat normal serum for 30 min at room temperature. The tissues were subsequently incubated with either primary antibody anti-mouse cardiac Troponin T (1:200, Thermo scientific), anti-mouse αSMA (1:100, Sigma), or anti-mouse PECAM (CD31) (1:100, BD Biosciences), for 1 hour at room temperature. Slides were washed three times in PBS, followed by incubation with Alexa Flour 488 conjugated secondary antibodies (1:500; Invitrogen) for 45 min at room temperature. Sections were counterstained with diamidino-2-phenylindole (DAPI) and photographed under a fluorescence microscope.

### Echocardiography Analysis

Echocardiography (Echo) was performed with Vevo2100 system (FujiFilm VisualSonics Inc.) with methods described previously [32]. Briefly, left ventricular cardiac function and structure were assessed from short axis B-mode and M-mode images at the level of the papillary muscles. M-mode recordings were used to measure ventricular wall thickness and chamber dimensions. VisualSonics Vevo 2100 V1.5.0 software (Visualsonics; Toronto, Canada) was used to measure echocardiographic parameters, including diastolic and systolic left ventricular internal diameter (LVID, short axis B-mode), anterior wall thickness (LVAW, short axis M-mode), posterior wall thickness (LVPW, short axis M-mode). Using left ventricular systolic and diastolic chamber dimensions, short-axis ejection fraction (LVEF) and fractional shortening (LVFS) were calculated within the software using standard formulas [32]. Heart rate (HR) and body temperature were monitored throughout image acquisition. All echocardiographic data acquisition and analysis were performed by two independent examiners blinded to the experimental groups. All quantitative data are expressed as mean ± SDEV. Differences between two groups were analyzed by Student’s *t* test using GraphPad Prism 6 software. *P*<0.05 was considered statistically significant.

## Results

### Generation of *Myh6* ^*MerCreMer/+*^ Knock-In Mouse Line

To achieve temporal inactivation of genes of interest in cardiomyocytes, we generated the *Myh6* ^*MerCreMer/+*^ knock-in mouse line by targeting the *Myh6* locus. A targeting vector containing the *MerCreMer-FRT-Neo-FRT* cassette flanked by homologous arms was electroporated into mouse ES cells. After homologous recombination, *MerCreMer-FRT-Neo-FRT* was inserted into the start codon of *Myh6* (Fig 1A). The recombinant bands 5.9-kb (5' end) and 4.3-kb (3' end) were amplified by long-range PCR with primers external to the arms and primers in the *MerCreMer-FRT-Neo-FRT* cassette (Fig 1B). *Myh6* ^*MerCreMer-Neo/+*^ mice derived from the positive ES cells were crossed with Flippase deleter mice [26] to obtain *Myh6* ^*MerCreMer/+*^ mice (Fig 1A). *Myh6* ^*MerCreMer/+*^ is a knock-in/knock-out allele for *Myh6* and those mice developed normally without any evident defects in appearance or behavior in Black Swiss background.

### Cardiac Cre Recombination Is Achieved in *Myh6* ^*MerCreMer/+*^ Mice After Tamoxifen Induction

**Fig 2:**
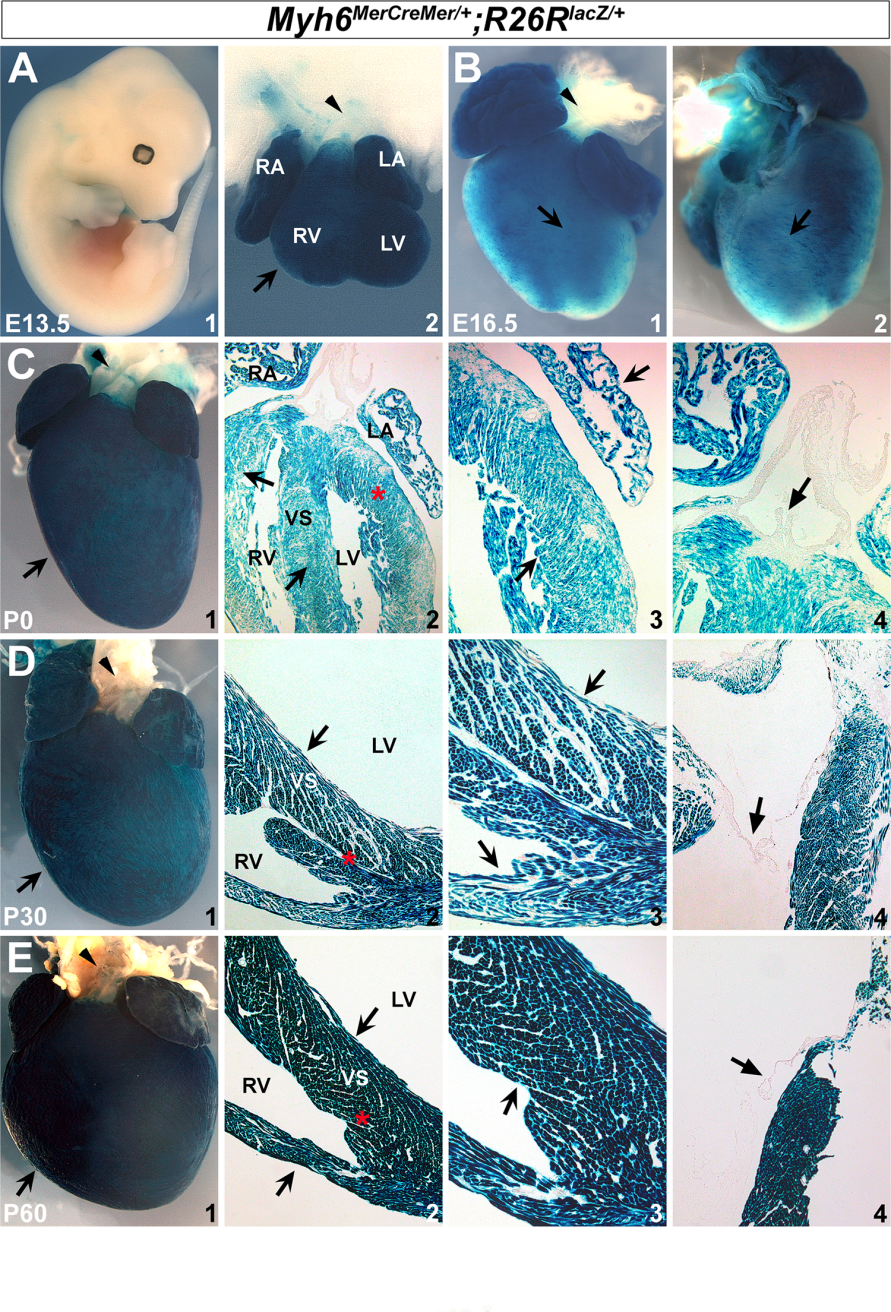
*Myh6*-driven Cre recombination is efficiently activated in heart after tamoxifen induction. X-gal staining revealed robust β-galactosidase expression in the embryonic and postnatal hearts (*R26R* ^*lacZ/+*^;*Myh6* ^*MerCreMer/+*^) after tamoxifen treatment. No X-gal positive cells were found in other regions on E13.5 embryos (A1). Notched arrows in A2/B1/C1/D1/E1 indicate positive X-gal staining in hearts. Arrowheads in A2/B1/C1/D1/E1 indicate pulmonary artery and aorta. Unnotched arrows in C4/D4/E4 indicate valves. C3/D3/E3 are high magnification images for C2/D2/E2 in the areas labeled by red asterisks. RA, right atrium; LA, left atrium; RV, right ventricle; LV, left ventricle; VS, ventricular septum.

**Fig 3:**
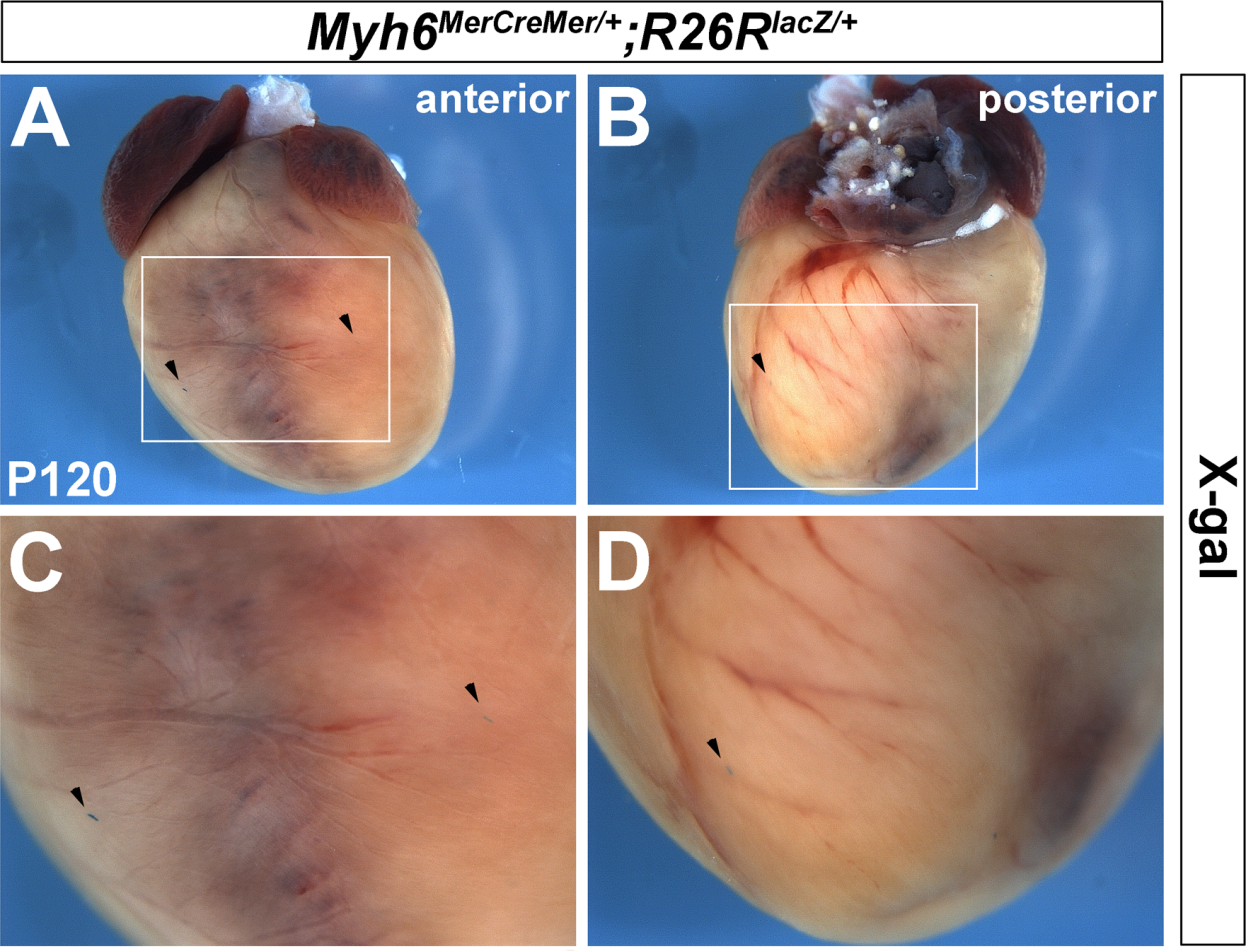
Recombination on *Myh6* ^*MerCreMer/+*^ hearts without tamoxifen. Very few X-gal positive cells were detected on *Myh6* ^*MerCreMer/+*^;*R26R* ^*lacZ/+*^ hearts without tamoxifen treatment. A is anterior view and B is posterior view of a 4-month-old heart (P120). C and D are high magnification of A and B in the square area, respectively. Arrowheads indicate X-gal positive cell.

To determine the efficiency and the specificity of Cre recombination in *Myh6* ^*MerCreMer/+*^ mice, we crossed *Myh6* ^*MerCreMer/+*^ mice with the *R26R* ^*lacZ/+*^ reporter line to generate *Myh6* ^*MerCreMer/+*^;*R26R* ^*lacZ/+*^ double heterozygous animals. β-galactosidase is expressed when Cre translocates from the cytoplasm to the nucleus after tamoxifen induction. To examine *Myh6*-mediated inducible Cre activity during gestation, tamoxifen was administered to pregnant dams with dosage of 0.05 mg/g body weight/day at embryonic days (E) 11.5, E14.5, and E16.5 by intraperitoneal injection for two consecutive days [29]. Whole embryos or hearts were harvested at E13.5, E16.5, and neonate (P0) to evaluate β-galactosidase activity, respectively. We observed robust X-gal staining in the hearts at E13.5, E16.5 and P0, but no staining was detected in other regions or organs (Fig 2A–2C). This revealed that tamoxifen effectively induces recombination in the hearts at embryonic stages. Furthermore, with three consecutive days of tamoxifen injection at 0.1 mg/g body weight/day, we were also able to detect robust X-gal staining on the postnatal hearts at P30 and P60 (Fig 2D–2E). Of note, X-gal staining was not detected in the aorta or pulmonary artery on *Myh6* ^*MerCreMer/+*^;*R26R* ^*lacZ/+*^ hearts at embryonic and adult stages (arrowheads in Fig 2A2,2B1,2C1,2D1 and 2E1). Further examination of cardiac sections revealed that β-galactosidase activity encompasses atrial and ventricular walls, as well as septal regions (notched arrows in Fig 2C2,2C3,2D2,2D3,2E2 and 2E3), but not valves (unnotched arrows in Fig 2C4,2D4 and 2E4). Moreover, very few X-gal positive cells were detected in *Myh6* ^*MerCreMer/+*^;*R26R* ^*lacZ/+*^ hearts at the adult stage when tamoxifen was not injected (Fig 3), suggesting that *Myh6* ^*MerCreMer/+*^ mice have very minimal Cre leakiness. Collectively, these observations indicated that the *Myh6* ^*MerCreMer/+*^ allele mediates highly efficient Cre-LoxP recombination in embryonic and postnatal hearts after tamoxifen induction.

### *Myh6* ^*MerCreMer/+*^ Introduces Specific Recombination in Cardiomyocytes

**Fig 4:**
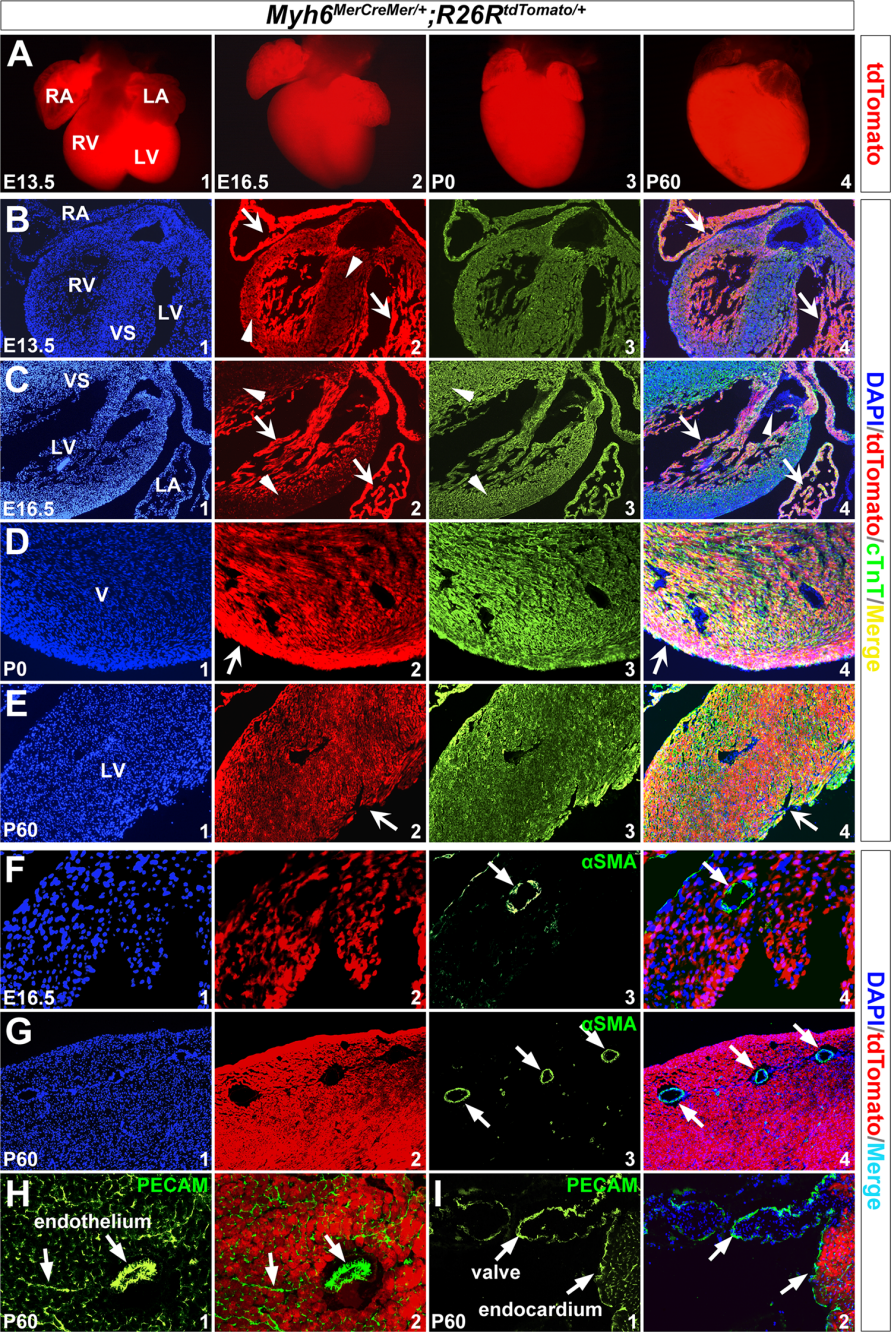
*Myh6* ^*MerCreMer/+*^ introduces specific recombination in cardiomyocytes. *R26R* ^*tdTomato/+*^ reporter line was used to trace *Myh6* lineage after tamoxifen induction. **(A)** tdTomato expression on the whole-mount hearts at E13.5-P60. **(B-I)** Immunostaining on transverse sections of hearts revealed tdTomato expression was co-localized with Tnnt2 (B-E), but was not co-localized with αSMA (F,G) or PECAM (H,I). Notched arrows in B2/C2/D2/E2 indicate tdTomato expression. Arrowheads in B2/C2 indicate lower level expression of tdTomato in VS and ventricular wall. Unnotched arrows in F,G indicate αSMA staining, and in H,I indicate PECAM staining. Arrowheads in C4 indicate valves. B4-G4 are merged images for B1/2/3-G1/2/3, respectively. H2 and I2 are merged images of H1 and I1 with corresponding tdTomato/DAPI images.

Next, we attempted to determine whether recombination mediated by *Myh6* ^*MerCreMer/+*^ is limited to cardiomyocytes. *R26R* ^*tdTomato/+*^ reporter mice were crossed with *Myh6* ^*MerCreMer/+*^ mice to obtain *Myh6* ^*MerCreMer/+*^;*R26R* ^*tdTomato/+*^ double heterozygous animals. Consistent with the X-gal staining results, high levels of tdTomato expression were observed on *Myh6* ^*MerCreMer/+*^;*R26R* ^*tdTomato/+*^ hearts at E13.5, E16.5, P0, and P60 following tamoxifen induction (Fig 4A). To examine whether the tdTomato-positive cells were cardiomyocytes, cardiac sections were made for immunofluorescence. We observed that at E13.5 and E16.5, even though robust tdTomato expression was detected in both atrial and ventricular walls, atrial fluorescent signals appear much stronger than that of ventricles. The trabeculated myocardium also displayed stronger fluorescence than the compact myocardium in the ventricles (arrows and arrowheads in Fig 4B2 and 4C2). This may reflect differential *Myh6* expression level in cardiac compartments at embryonic stages as previously described [33]. From birth to adulthood, tdTomato signals were evenly distributed in the ventricle (notched arrows in Fig 4D2 and 4E2). We further performed immuostaining with anti-cardiac troponin T (cTnT/Tnnt2, marker for cardiomyocytes), anti-alpha smooth muscle actin (αSMA, marker for vascular smooth muscle cells), and anti-PECAM (marker for endothelial cells) [34–37]. The results showed that tdTomato was fully co-localized with cTnT in the heart at E16.5-P60 (notched arrows in Fig 4B4,4C4,4D4 and 4E4), but not with αSMA or PECAM in the coronary smooth muscle cells (unnotched arrows in Fig 4F4 and 4G4) or endothelial/endocardial cells (unnotched arrows in Fig 4H2 and 4I2). Additionally, tdTomato was not detected in valves (arrowheads in Fig 4C4 and 4I2). This indicates that *Myh6* ^*MerCreMer/+*^ specifically drives Cre recombination in cardiomyocytes.

### *Myh6* ^*MerCreMer/+*^ Hearts Display Normal Structure and Function before and after Tamoxifen Induction

**Fig 5:**
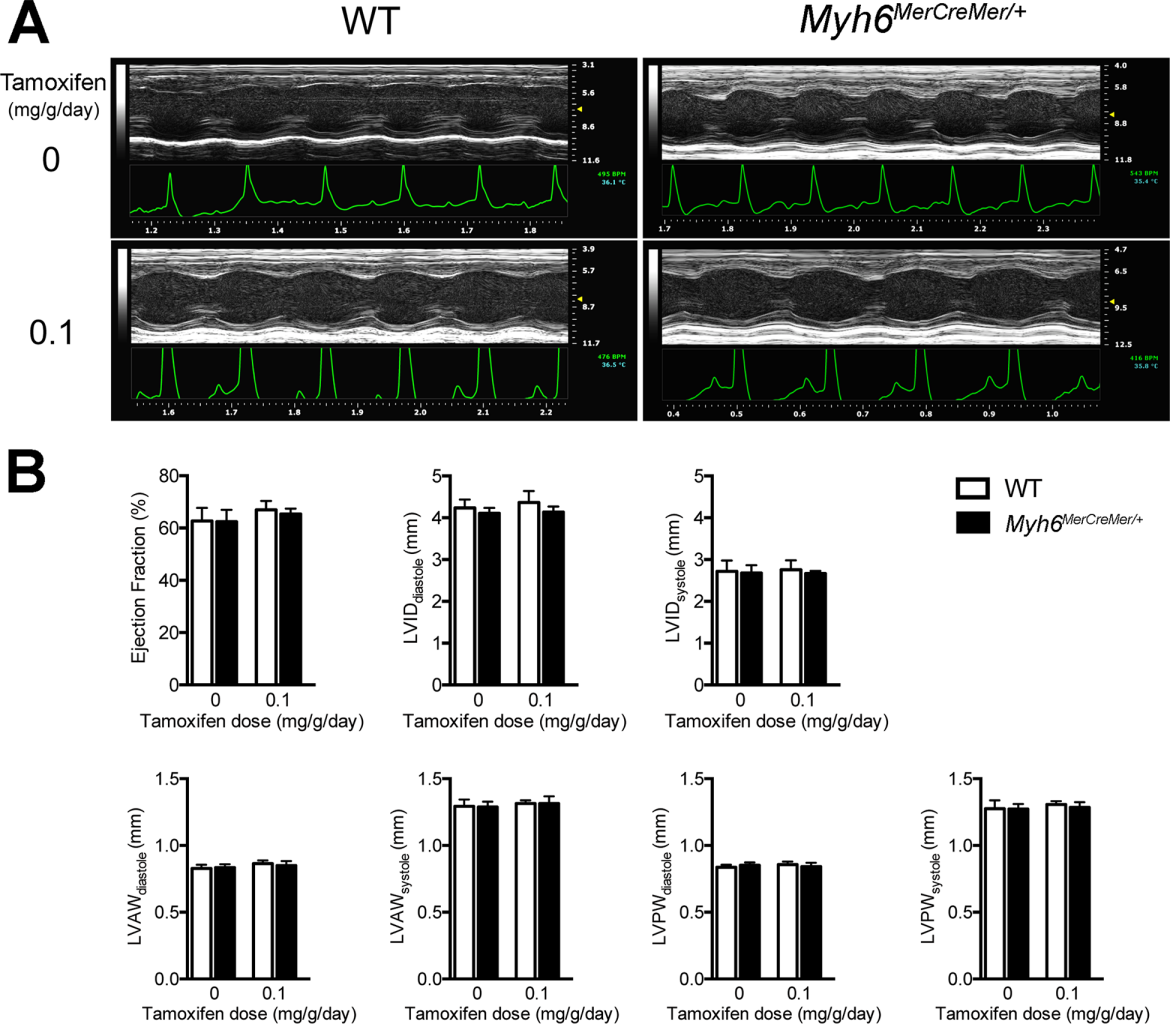
Normal cardiac function and dimensions after tamoxifen induction in *Myh6* ^*MerCreMer/+*^ mice. **(A)** M-mode echocardiography of the same *Myh6* ^*MerCreMer/+*^ and wild type littermate mouse at baseline and five weeks after tamoxifen administration. **(B)** Quantified indices of left ventricular function and structure before and after Cre induction. Tamoxifen was administered at 0.1 mg/g body weight/day. Ejection fraction, EF; left ventricular internal diameter, LVID; left ventricular anterior wall, LVAW; left ventricular posterior wall, LVPW. All values were plotted as mean ± STDEV.

**Fig 6:**
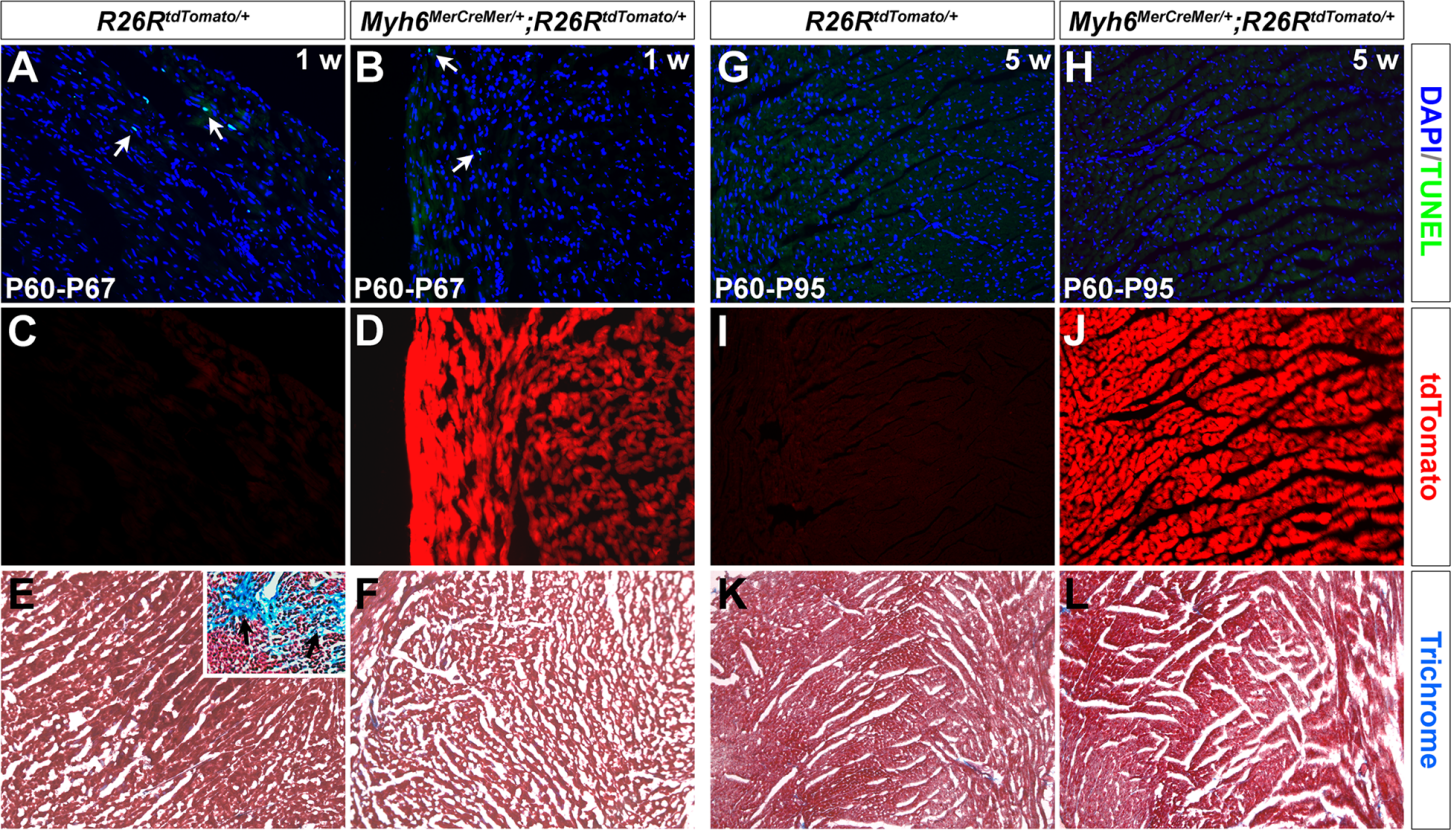
Tamoxifen has little effect on *Myh6* ^*MerCreMer/+*^ hearts. Apoptosis and fibrosis assays on *Myh6* ^*MerCreMer/+*^ mouse hearts in one week (A-F) and five weeks (G-L) after tamoxifen injection (0.01 mg/g body weight for 3 days). A few apoptotic cells were observed on both *R26R* ^*tdTomato/+*^ and *Myh6* ^*MerCreMer/+*^;*R26R* ^*tdTomato/+*^ hearts after one week (arrows in A,B, P60→P67), and very few detected after five weeks (G,H, P60→P95). Tamoxifen mediates robust recombination on *Myh6* ^*MerCreMer/+*^;*R26R* ^*tdTomato/+*^ hearts (D,J), but not on *R26R* ^*tdTomato/+*^ control hearts (C,I). Myocardial fibrosis was not detected on *R26R* ^*tdTomato/+*^ or *Myh6* ^*MerCreMer/+*^;*R26R* ^*tdTomato/+*^ hearts by trichrome staining after one (E,F) and five weeks (K,L). Image in the upright corner of E is from an unrelated study, showing positive trichrome staining and cardiac fibrosis (arrows). This staining was performed in parallel with sample in E.

**Fig 7:**
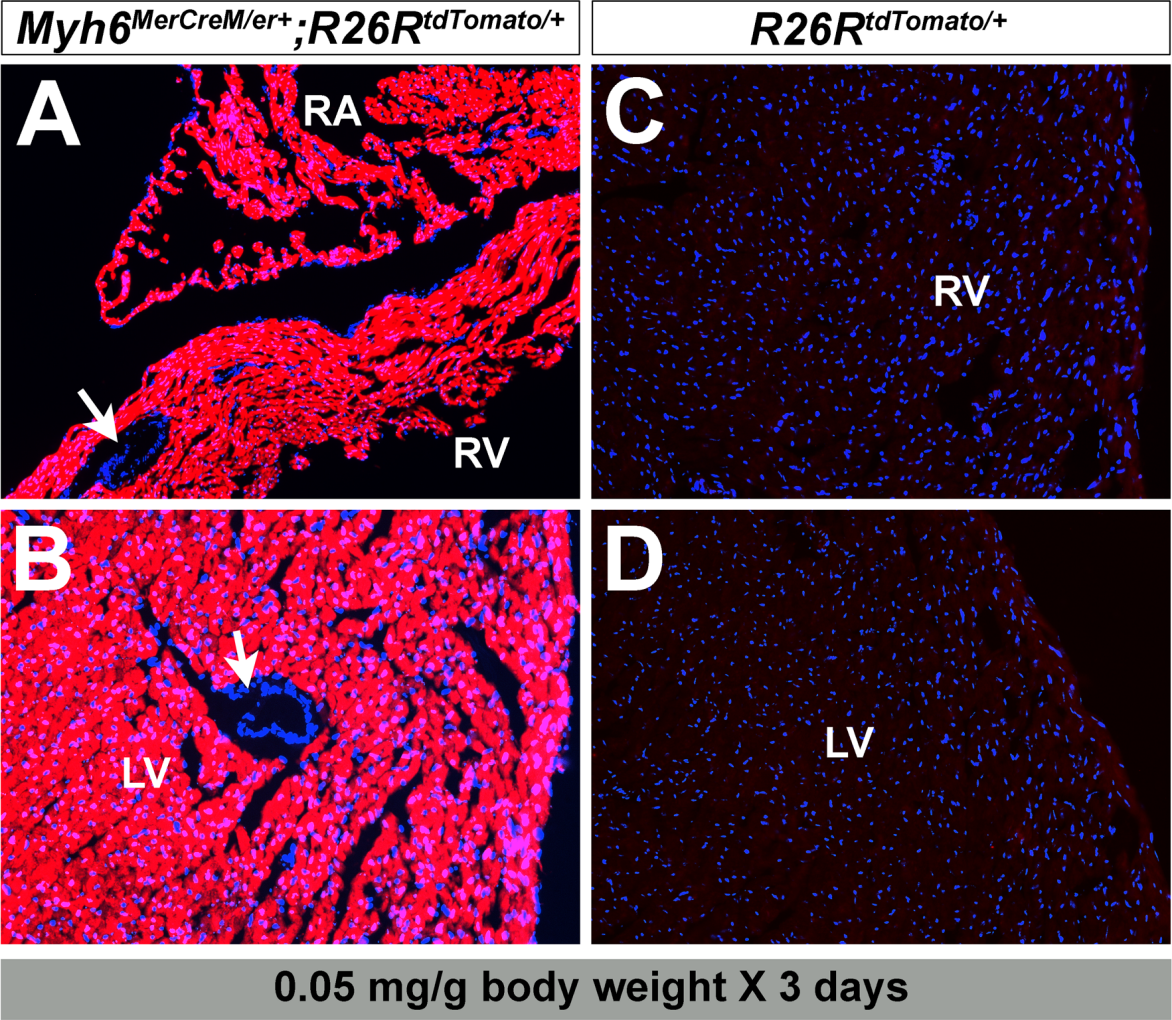
Lower dosage of tamoxifen mediates sufficient recombination on adult *Myh6* ^*MerCreMer/+*^ hearts. Administration of tamoxifen at 0.05 mg/g body weight for 3 days also introduces robust tdTomato expression in *Myh6* ^*MerCreMer/+*^;*R26R* ^*tdTomato/+*^ mouse hearts (A,B). No recombination occurs in the control hearts (*R26R* ^*tdTomato/+*^, C,D). Arrows in A,B indicate coronary vessels.

Given that *Myh6* ^*MerCreMer/+*^ is a knock-in/knock-out allele for *Myh6*, it is important to know whether the cardiac function and structure were adversely affected by *MerCreMer* insertion. We performed transthoracic echocardiography on *Myh6* ^*MerCreMer/+*^ and wild type littermate mice at P60-90 (n = 10 for each group). Left ventricular short-axis measurements showed no change in cardiac structure and function between *Myh6* ^*MerCreMer/+*^ and wild type littermate mice (Fig 5A and S1 and S2 Videos). There was no significant difference in cardiac chamber dimensions, wall thicknesses, fractional shortening, or ejection fraction in *Myh6* ^*MerCreMer/+*^ mice when compared to their wild type littermates (Fig 5B, Table 1), suggesting *MerCreMer* insertion into the *Myh6* start codon had no effect on cardiac development and function. Furthermore, to determine whether tamoxifen administration had any impact on cardiac function, we performed echocardiography on *Myh6* ^*MerCreMer/+*^ and wild type littermates five weeks after the final injection (0.1 mg/g body weight/day for 3 days). No significant difference was found between *Myh6* ^*MerCreMer/+*^ and littermate controls (n = 6 for each group, Fig 5, Table 1 and S3 and S4 Videos). Subsequent TUNEL and trichrome staining demonstrated that tamoxifen does not lead to myocardial apoptosis or fibrosis on *Myh6* ^*MerCreMer/+*^ hearts one and five weeks after injection (Fig 6). In addition, we attempted to determine the minimum effective tamoxifen dosage to minimize any potential cardiac toxicity. With 0.05 mg/g body weight/day for three days, the adult *Myh6* ^*MerCreMer/+*^ hearts still exhibited sufficient recombination one month after tamoxifen injection (Fig 7).

**Table 1:** Transthoracic echocardiography of *Myh6* ^*MerCreMer/+*^ mice.

TMX dose (mg/g/day)	Genotype	Number (n)	HR (BPM)	LVID,s (mm)	LVID,d (mm)	FS (%)	LVAW,d (mm)	LVAW,s (mm)	LVPW,d (mm)	LVPW,s (mm)	EF (%)
**Baseline**	Wild type	10	465 ± 48	2.72 ± 0.26	4.24 ± 0.20	35.8 ± 4.6	0.83 ± 0.03	1.29 ± 0.05	0.84 ± 0.02	1.28 ± 0.06	62.7 ± 5.0
	*Myh6* ^*MerCreMer/+*^	10	482 ± 45	2.68 ± 0.19	4.11 ± 0.13	34.8 ± 4.7	0.83 ± 0.03	1.29 ± 0.04	0.85 ± 0.02	1.27 ± 0.04	62.4 ± 4.6
	*p*-value		0.41	0.67	0.10	0.62	0.59	0.79	0.09	0.92	0.89
**0.1**	Wild type	6	441 ± 44	2.75 ± 0.23	4.37 ± 0.27	36.9 ± 2.6	0.86 ± 0.02	1.31 ± 0.02	0.86 ± 0.02	1.31 ± 0.02	67.0 ± 3.4
	*Myh6* ^*MerCreMer/+*^	6	421 ± 22	2.66 ± 0.06	4.14 ± 0.13	35.5 ± 1.6	0.85 ± 0.03	1.31 ± 0.05	0.84 ± 0.03	1.29 ± 0.04	65.4 ± 2.0
	*p*-value		0.36	0.37	0.09	0.29	0.40	1.00	0.34	0.27	0.35

Normal left ventricular dimensions and function of *Myh6* ^*MerCreMer/+*^ mice in comparison to wild type controls. TMX, Tamoxifen; Heart rate, HR; left ventricular internal diameter, diastole, LVID,d; LVID, systole, LVID,s; fractional shortening, FS; left ventricular anterior wall, systole, LVAW,s; LVAW, diastole, LVAW,d; left ventricular posterior wall, diastole, LVPW,d; LVPW, systole, LVPW,s; ejection fraction, EF. Groups were compared using Student’s *t*-test. *P* < 0.05 was considered significant. All values were expressed as mean ± STDEV.

## Discussion

In this study, we described generation and characterization of a new *Myh6* ^*MerCreMer/+*^ knock-in mouse model. *Myh6* ^*MerCreMer/+*^ mice develop normally without cardiac functional defects. Short-term tamoxifen treatment resulted in efficient Cre recombination in cardiomyocytes. This new *Myh6* ^*MerCreMer/+*^ animal is a useful tool for deletion of genes of interest in myocardium with Cre-LoxP technology at desirable stages.

A few mouse lines were created previously for inducible genetic deletion in myocardium with tetracycline and *MerCreMer* systems [11, 12]. The tetracycline-inducible system requires two transgenes: a reverse tetracycline-controlled transactivator (rtTA) directed by a rat *cTnT* promoter and a Cre recombinase driven by a tetracycline responsive promoter (TetO), thereby making breeding scenarios complicated [11]. Another limitation of the tetracycline-inducible system is potential leakiness [38]. The *α-MHC-MerCreMer* transgenic mouse line was generated and had been used widely for gene inactivation in the myocardium [12, 39–42]. However, a few studies showed that the *α-MHC-MerCreMer* mouse line displayed myocardial fibrosis and cardiac dysfunction due to Cre-induced DNA damage and myocardial apoptosis after tamoxifen induction [15–21]. In this new *Myh6* ^*MerCreMer/+*^ mouse, Cre recombination is strongly activated within cardiomyocytes following tamoxifen induction. This animal exhibited relatively good tolerance to tamoxifen (no myocardial fibrosis or apoptosis) and displayed normal cardiac structure and function after appropriate induction. Moreover, a low dosage of tamoxifen (0.05 mg/g body weight for 3 days) also introduces robust and specific recombination in the cardiomyocytes at adult stage.

*Myh6* ^*MerCreMer/+*^ is a heterozygous null for *Myh6* (*Myh6* ^*+/−*^). *Myh6* ^*+/−*^ animals were shown to have cardiac functional defects with sarcomeric structural alterations and fibrosis [43]. However, by performing trichrome staining and echocardiography, we did not detect effects on *Myh6* ^*MerCreMer/+*^ hearts (2–3 months old, n = 10, Fig 5, Table 1, and S1 and S2 Videos). The discrepancy could be due to the difference in the gene targeting strategy and/or a genetic divergence between these animals: in *Myh6* ^*MerCreMer/+*^ mice, the *MerCreMer-FRT-Neo-FRT* cassette was inserted into the ATG locus and the *Neomycin* sequence was removed by Flippase deleter mice. In the *Myh6* ^*+/−*^ mice examined by Jones *et al* [43], the *pgk-Neo-polyA* cassette was targeted into the *Myh6* locus with a deletion of an approximately 2-kb fragment of the *Myh6* gene. The deleted sequence includes the first three exons, the 5' untranslated region, and the initiating methionine codon [43]. It is uncertain whether the existing *Neomycin* cassette in this strong myocardial locus has any negative effects on cardiac function. Moreover, it is important to note that the physiologic and pathologic phenotypes in *Myh6* ^*+/−*^ mice are not completely penetrant [43]. *Myh6* ^*MerCreMer/+*^ animals in this study are in hybrid background (Black Swiss), and genetic and epigenetic variations could potentially be important factors for *Myh6* ^*+/−*^ heart function [43]. In the future, it will be of interest to determine whether the inbred background of *Myh6* ^*MerCreMer/+*^ mice has any impact on their cardiac performance.

As mentioned before, tamoxifen injection into *α-MHC-MerCreMer* transgenic line could lead to severe toxicity to the heart [15–21]. Mice with three doses of tamoxifen at 0.03–0.09 mg/g body weight/day displayed cardiac fibrosis and dysfunction, with 10–50% mortality within one week [20, 21]. In this study, we found that with three doses of tamoxifen at 0.1 mg/g body weight/day, the *Myh6* ^*MerCreMer/+*^ mice appeared normal in cardiac function and structure and no lethality was observed. No myocardial fibrosis or apoptosis was found in *Myh6* ^*MerCreMer/+*^ mice after one and five weeks of administration (Figs 4 and 5). This may be explained by the genetic difference between *α-MHC-MerCreMer* and *Myh6* ^*MerCreMer/+*^ mice. *α-MHC-MerCreMer* is a transgenic line and each myocardial cell may have multiple copies of *MerCreMer* (note each *MerCreMer* cassette has its own *α-MHC* promoter) [44]. In contrast, *Myh6* ^*MerCreMer/+*^ knock-in animals only have one copy of *MerCreMer* in their genome. Therefore, *MerCreMer* expression in *α-MHC-MerCreMer* myocardial cells might be much higher than that in *Myh6* ^*MerCreMer/+*^ myocardial cells. Under certain dosage of tamoxifen induction (e.g., 0.1 mg/g body weight/day for three days), the high level *MerCreMer* expression may lead to excessive amount of Cre translocation to nuclei which in turn may induce DNA damage and cell death in the cardiomyocytes. *Myh6* ^*MerCreMer/+*^ cardiomyocytes have lower *MerCreMer* expression and do not have a large amount of Cre translocation under this dosage.

The major application of this new *Myh6* ^*MerCreMer/+*^ mouse model will be the temporal disruption of genes of interest in cardiomyocytes *in vivo*. Given that almost all the myocardial cells robustly express Cre after tamoxifen induction, this inducible Cre mouse line can also be applied to determine myocardial lineage during development and after cardiac injury.

## Supporting Information

S1 Video Echocardiography analysis of wild type mouse without tamoxifen treatment. (AVI)

S2 Video Echocardiography analysis of *Myh6* ^*MerCreMer/+*^ mouse without tamoxifen treatment. (AVI)

S3 Video Echocardiography analysis of wild type mouse with tamoxifen treatment. (AVI)

S4 Video Echocardiography analysis of *Myh6* ^*MerCreMer/+*^ mouse with tamoxifen treatment. (AVI)
